# Supplementary material for: Assessing the bread-leavening ability of wild yeasts isolated from selected fruits collected from local markets
Source: PLoS One. 2025 May 20;20(5):e0324252. doi: 10.1371/journal.pone.0324252 (PMC12091880; doi:10.1371/journal.pone.0324252)
Supplement: Attachment — Submitted filename: Reviewers comments.docx [file pone.0324252.s007.docx]

**Reviewer’s Comments on Assessing the Bread-Leavening Ability of Wild Yeasts Isolated from Selected Fruits Collected from Local Markets**

This study effectively explores the potential of wild yeast isolates from fruits for dough leavening, showing promising results in CO2 production and non-H2S generation. The research is well-structured, with thorough biochemical, cultural, and morphological analysis, and highlights the superiority of co-culture systems for industrial application.

The abstract succinctly outlines the research objectives, methodology, results, and potential applications, providing a clear and structured summary. Keywords are relevant and help highlight the primary focus areas of the study, improving its discoverability. Highlighting the baking applications adds practical value to the study, making it relevant to industry and academia.

The introduction section effectively covers the historical and practical importance of *Saccharomyces cerevisiae* in bread baking, offering both global and local (Ethiopian) perspectives. Emphasizing on leavening agents and their role provides a solid basis for the study. The introduction also highlights the reliance on imported baker’s yeast and the associated economic impact, justifying the need for local alternatives. However, it lacks a clear research gap, and the results and discussion sections would benefit from more quantitative data and deeper analysis.

The conclusion summarizes the findings but could offer more specificity and a broader discussion. Overall, the study makes a valuable contribution but requires some improvements in clarity and depth. The emphasis on wild yeast isolates as potential alternatives to commercial yeast for bread-making is a significant contribution to the field. Also the comparison between the wild yeast isolates and commercial yeast adds depth and showcases the practical implications of the research. Optimal conditions (temperature, pH, glucose, NaCl concentration) for yeast growth are well-discussed, showing the practical applicability of the findings.

**Suggestions for improvement:**

- The abstract mentions superior performance of AAUGr5 over commercial yeast but does not quantify or provide a benchmark. A brief mention of the percentage improvement or comparative analysis would add clarity.
- The term "tentatively identified" suggests a lack of definitive confirmation. Consider stating whether molecular techniques (e.g., sequencing) were used for more accurate identification.
- Split longer sentences into two or more for better readability
- The statement “Yeast species utilize the abundant sugar found in fruits and can be isolated from these sources” is repeated in close proximity in introduction section. Consolidate this information for clarity and conciseness.
- The final sentences of introduction mention "potential commercial-scale production" but doesn’t tie it back to the economic benefits or feasibility. Adding a sentence about how this could reduce Ethiopia’s reliance on imports would strengthen the argument.
- Some sentences are overly long and complex. Simplify them for better readability.
- Specify the exact conditions of storage (e.g., temperature, covered/uncovered).
- Mention if the method for active dry baker's yeast (Saf instant) preparation was standardized before use.
- Provide more detail on colony selection criteria (e.g., size, color, morphology).
- Mention how many colonies were selected for purification and why.
- Describe the specific cultural traits examined (e.g., colony size, shape, color) in culture identification.
- Indicate how many yeast isolates were examined and if any replicates were performed.
- Mention the rationale behind choosing specific pH, temperature, glucose, and NaCl concentrations.
- In discussion section, some points are heavily dependent on previously published studies without a deeper explanation of how this study advances the field.
- The discussion does not address limitations of the study, such as sample size, possible variability in wild yeast performance, or challenges in scaling up for industrial use.
- Include a discussion on how the findings could impact the baking industry or contribute to sustainable production practices.
- Reflect on why the wild yeast isolates performed similarly or better than commercial yeast.
