# Supplementary material for: Assessing the bread-leavening ability of wild yeasts isolated from selected fruits collected from local markets
Source: PLoS One. 2025 May 20;20(5):e0324252. doi: 10.1371/journal.pone.0324252 (PMC12091880; doi:10.1371/journal.pone.0324252)
Supplement: Attachment — Submitted filename: Response to Reviewers.docx [file pone.0324252.s009.docx]

**Authors reply to the academic editor and reviewer's comments**

**Dear Editor and Reviewers;**

The manuscript entitled “Assessing the bread-leavening ability of wild yeasts isolated from selected fruits collected from local markets” has been revised in response to the academic editor and reviewer’s comments. We have found that all the comments are interesting and important to improve the manuscript further. We appreciate and forward our gratitude to the academic editor and reviewers for their exciting comments. This file lists the responses to each point raised by the reviewers. The corrections in the revised manuscript have been highlighted in red. In addition, we have made some language improvements and technical error corrections, which are not highlighted in the manuscript.

**Reviewers' comments:**

**Reviewer #1:** This manuscript focuses on evaluating the bread fermentation ability of wild yeasts isolated from specific fruits. It excels in several areas but also has room for improvement.

The motivation for the study is reasonable and sustainable. In Ethiopia, the baking industry heavily relies on imported yeast and baking powder, which is costly, providing a clear incentive to explore alternative sources of yeast. Additionally, the issue of hydrogen sulfide production by commercial yeasts, which negatively affects bread quality, makes the search for more suitable yeast strains particularly necessary. The research objectives are well-defined and closely related to these practical issues, aiming to identify efficient wild yeast strains that could improve the bread-making process.
The experimental design is technically sound and carefully planned. Various fruit samples, including avocado, banana, grape, mango, orange, papaya, and pineapple, were collected from local markets. The isolation of wild yeasts followed a standard procedure, where yeast cells were obtained by serial dilution and cultured on potato dextrose agar (PDA) plates containing chloramphenicol. The selection process based on CO₂ production and the absence of hydrogen sulfide is a reasonable approach for identifying potentially useful yeast strains, aligning well with the study’s objectives. Subsequent identification steps, including culturing, morphological, and biochemical analyses, provide a comprehensive understanding of the selected yeast strains. Using the Dutch DSM baking strain as a standard reference is a common and reliable practice, and previous studies have also used it as a control, so its credibility is likely not in question. The use of SPSS for data analysis is also appropriate.

However, there are some areas for improvement. In the methodology section, although the experimental steps are described in detail, some of the references for material processing methods are somewhat outdated. Although my research focus is not on yeast extraction, in the rapidly evolving field of microbiology, the authors should clarify whether there are more suitable processing methods or whether these methods are considered the gold standard. This would enhance the accuracy and efficiency of the research process.

**Response:** We appreciate the reviewer’s concern. However, we were unable to use recent technologies like DNA sequencing for the accurate identification of the yeast isolates due to a lack of funds. Moreover, we believe that the methods used in our study are the gold standard and we will use recent methods like DNA sequencing and cloning the desired gene to enhance their leavening potential in our future advanced studies.

Another issue is data availability. Given the promising results presented in the manuscript, the authors could point out appropriate ways to access the raw data.

**Response:** Thank you. We will make our raw data accessible online and will be submitted via the journal submission system.

Finally, the manuscript spends a considerable amount of space on the selection, identification, and characterization of yeast strains. However, in evaluating fermentation ability, focusing only on the dough's volume change seems somewhat limited. Bread quality is a multidimensional attribute, and other aspects such as texture, color, and flavor also play a crucial role in determining its overall acceptability. These aspects might also be suitable for assessing the fermentation ability of yeast strains, providing a more comprehensive evaluation of their performance. For example, including images from different stages of fermentation could provide valuable visual information and enhance the clarity of the results.

**Response:** We are so happy for these critical comments. As stated in the manuscript our objective was the evaluation of wild yeast for their bread leavening capacity under laboratory scale conditions. In this regard, we tried to use various parameters to identify and screen those 88 yeast isolates and finally, we came up with 3 wild yeast isolates that are capable of producing high CO_2_ as observed in the Durham tube. However, there was a possibility of evaluating other attributes of the isolates as stated by the reviewer, and will be considered for our future studies.

While the study shows potential, these possible flaws should be addressed. I believe this research will not only contribute to the field of yeast studies but could also impact the baking industry in Ethiopia and other regions. Overall, it is a good study.

**Response:** Thank you so much for encouraging. This means a lot to us and it will help us to do further advanced work.

**Reviewer #2:** This study effectively explores the potential of wild yeast isolates from fruits for dough leavening, showing promising results in CO_2_ production and non-H_2_S generation. The research is well-structured, with thorough biochemical, cultural, and morphological analysis, and highlights the superiority of co-culture systems for industrial application. However, Minor revision is required to make the manuscript more effective.

**Response:** We are so thankful for the reviewer's suggestion and maximum efforts were utilized to improve the status of the manuscript based on the comments given from the attachment and now the revised manuscript is improved to a great extent.
