# Supplementary material for: Assessing the bread-leavening ability of wild yeasts isolated from selected fruits collected from local markets
Source: PLoS One. 2025 May 20;20(5):e0324252. doi: 10.1371/journal.pone.0324252 (PMC12091880; doi:10.1371/journal.pone.0324252)
Supplement: Attachment — Submitted filename: Response to Reviewers 2.docx [file pone.0324252.s010.docx]

**Authors reply to the academic editor and reviewer's comments**

**Dear Editor and Reviewers;**

The manuscript entitled “Assessing the bread-leavening ability of wild yeasts isolated from selected fruits collected from local markets” has been revised in response to the academic editor and reviewer’s comments. We have found that all the comments are interesting and important to improve the manuscript further. We appreciate and forward our gratitude to the academic editor and reviewers for their exciting comments. This file lists the responses to each point raised by the reviewers. The corrections in the revised manuscript have been highlighted in red. In addition, we have made language improvements and technical error corrections, which are not highlighted in the manuscript. Moreover, we did not cite references that have been retracted, and all the references cited are complete and correct. But reference number [10] is exceptional as it is simply a reference that indicates data from the central statistical agency of Ethiopia.

**Reviewers' comments:**

1. If the authors have adequately addressed your comments raised in a previous round of review and you feel that this manuscript is now acceptable for publication, you may indicate that here to bypass the “Comments to the Author” section, enter your conflict of interest statement in the “Confidential to Editor” section, and submit your "Accept" recommendation.

**Reviewer #1**: All comments have been addressed

**Reviewer #3**: (No Response)

**Response**: We love and appreciate the reviewer's concern about the improvements of the manuscript as per their previous comments.

2. Is the manuscript technically sound, and do the data support the conclusions?
The manuscript must describe a technically sound piece of scientific research with data that supports the conclusions. Experiments must have been conducted rigorously, with appropriate controls, replication, and sample sizes. The conclusions must be drawn appropriately based on the data presented.

**Reviewer #1**: Yes

**Reviewer #3**: (No Response)

**Response**: Thank you so much.

3. Has the statistical analysis been performed appropriately and rigorously?

**Reviewer #1**: Yes

**Reviewer #3**: (No Response)

**Response**: Thank you so much.

4. Have the authors made all data underlying the findings in their manuscript fully available?
The [PLOS Data policy](http://track.editorialmanager.com/CL0/http:%2F%2Fwww.plosone.org%2Fstatic%2Fpolicies.action%23sharing/1/010f0195c67dff11-d25db938-05c8-4fbc-b431-689d804672e0-000000/y4A2vs7l-s0veSR9_dsMMMzeHvtVdgHF11YMDQi3Zfg=203) requires authors to make all data underlying the findings described in their manuscript fully available without restriction, with rare exception (please refer to the Data Availability Statement in the manuscript PDF file). The data should be provided as part of the manuscript or its supporting information, or deposited to a public repository. For example, in addition to summary statistics, the data points behind means, medians and variance measures should be available. If there are restrictions on publicly sharing data—e.g. participant privacy or use of data from a third party—those must be specified.

**Reviewer #1**: Yes

**Reviewer #3**: (No Response)

**Response**: Thank you so much.

5. Is the manuscript presented in an intelligible fashion and written in standard English?
PLOS ONE does not copyedit accepted manuscripts, so the language in submitted articles must be clear, correct, and unambiguous. Any typographical or grammatical errors should be corrected at revision, so please note any specific errors here.

**Reviewer #1**: Yes

**Reviewer #3**: (No Response)

**Response**: Thank you so much.

6. Review Comments to the Author

Please use the space provided to explain your answers to the questions above. You may also include additional comments for the author, including concerns about dual publication, research ethics, or publication ethics. (Please upload your review as an attachment if it exceeds 20,000 characters)

**Reviewer #1**: The authors have made remarkable improvements to the manuscript. The structural optimization has significantly enhanced the overall readability and logical coherence, guiding readers more smoothly through the research process from start to finish. By eliminating potentially misleading expressions, the clarity of the content has been greatly improved, ensuring that the scientific information is accurately conveyed. The expanded discussion on the impact on Ethiopian agriculture adds a practical and far - reaching dimension to the research, demonstrating the potential real - world implications of the study. Although some issues raised in the previous review remain unaddressed, likely due to the current research scale, this does not detract from the article's innovation and advantages. The exploration of wild yeasts for bread - leavening is novel and holds great promise. Overall, the article now substantially meets the basic requirements of PLOS One for published manuscripts, and it is expected that the authors may further address these issues in future research endeavors.

**Response**: We love the encouragement and such the lovely feedback from the reviewer. We just tried our best to improve the status of the manuscript, and it has been improved to a great extent. Moreover, the study is crucial mainly for developing countries like Ethiopia, as importing bakers’ yeast is highly devastating for the country’s economy. Similarly, we will address all the issues raised by the reviewer in our future studies.

**Reviewer #3:** Kindly recheck language and grammar issues to improve clarity, coherence, and overall professionalism of the manuscript.

**Response**: Dear reviewer, we appreciate your concern, and we have tried our best to improve the over quality of the manuscript and the manuscript has been improved to the standard in this regard.

7. PLOS authors have the option to publish the peer review history of their article ([what does this mean?](https://track.editorialmanager.com/CL0/https:%2F%2Fjournals.plos.org%2Fplosone%2Fs%2Feditorial-and-peer-review-process%23loc-peer-review-history/1/010f0195c67dff11-d25db938-05c8-4fbc-b431-689d804672e0-000000/knlLb0sVrS-MpmQ5Fe7vjwlXTxFEZOqZtTcvIhy6zcA=203)). If published, this will include your full peer review and any attached files.
If you choose “no”, your identity will remain anonymous but your review may still be made public.
**Do you want your identity to be public for this peer review?** For information about this choice, including consent withdrawal, please see our [Privacy Policy](https://track.editorialmanager.com/CL0/https:%2F%2Fwww.plos.org%2Fprivacy-policy/1/010f0195c67dff11-d25db938-05c8-4fbc-b431-689d804672e0-000000/w29R59WWOUD3GBIM8JHT32BGK8Uq0JyXGIqIPyN-RN4=203).

**Reviewer #1**: No

**Reviewer #3**: No

**Response**: Thank you so much.
